# The Relationship Between Impulsivity and Neuropsychological Profile in Multiple Sclerosis

**DOI:** 10.1002/brb3.71865

**Published:** 2026-11-13

**Authors:** Mariachiara Gaita, Antonio Costanzo, Valentina Torchia, Ylenia Crocetto, Luigi Trojano, Gabriella Santangelo, Stefania Miniello, Simona Raimo

**Affiliations:** ^1^ Department of Psychology University of Campania “Luigi Vanvitelli” Caserta Italy; ^2^ Department of Medical and Surgical Sciences University “Magna Graecia” of Catanzaro Catanzaro Italy; ^3^ Department of Health Sciences “Magna Graecia” University of Catanzaro Catanzaro Italy; ^4^ Neurology and Stroke Unit “Sant'Anna e San Sebastiano” Hospital Caserta Italy

**Keywords:** behavioral disorders, executive function, impulsivity, intra‐individual cognitive variability, multiple sclerosis

## Abstract

**Introduction:**

Impulsivity is increasingly recognized as a clinically relevant behavioral feature in people with multiple sclerosis (PwMS), but its neuropsychological correlates and the possible influence of intra‐individual cognitive variability (IICV), an index of fluctuation in performance across tasks or within testing sessions, remain unclear. The aim of this study was to investigate the relationship between impulsivity and neuropsychological profile in PwMS and to determine whether these associations persist after accounting for IICV.

**Methods:**

Fifty‐four PwMS underwent an assessment of cognitive abilities, including the Brief Repeatable Battery of Neuropsychological Tests. Behavioral measures included the Barratt Impulsiveness Scale (BIS‑11), Beck Depression Inventory‑II (BDI‐II), Dimensional Apathy Scale (DAS), and Self‑Awareness Questionnaire (SAQ). IICV indices were derived from performance across memory, executive, and attention/processing speed using maximum discrepancy and intra‐individual standard deviation metrics. Spearman correlations and hierarchical regression models were used to examine relations between impulsivity and other neuropsychological measures.

**Results:**

Higher non‐planning impulsivity was associated with poorer processing speed (SDMT: rho = −0.430, *p* = 0.001) and higher apathy (DAS Total: rho = 0.546, *p* < 0.001). Attentional impulsivity was associated with higher interoceptive sensibility (SAQ Total: rho = 0.405, *p* = 0.002), apathy (DAS Total: rho = 0.442, *p* = 0.001; DAS Executive: rho = 0.467, *p* < 0.001), and depressive symptoms (BDI‐II: rho = 0.570, *p* < 0.001). Non‐planning impulsivity remained an independent predictor of SDMT performance after adjustment for attention/processing‐speed IICV (*β* = −0.363, *p* = 0.004). No significant associations were found for motor impulsivity.

**Conclusions:**

In PwMS, non‐planning and attentional impulsivity appear to be associated with distinct cognitive and behavioral alterations, independently of IICV. Impulsivity could represent a clinically informative marker of top‐down control dysfunction and a potential target for cognitive and behavioral interventions.

## Graphical abstract

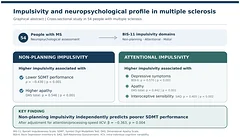

## 1 Introduction

Multiple sclerosis (MS) is a chronic demyelinating disorder of the central nervous system, characterized by sensory and motor disturbances, as well as cognitive (40%–70%) and behavioral changes that significantly affect quality of life (Rocca et al. 2015; Ruano et al. 2017). Cognitive and behavioral changes are being increasingly recognized as crucial clinical characteristics, as they may help identify people with MS (PwMS) who are at greater risk of further cognitive decline (Raimo, Spitaleri, et al. 2020). Although the clinical implications of some behavioral alterations, such as apathy (Raimo, Trojano, et al. 2020; Raimo et al. 2022) and depression (Feinstein et al. 2014; Sullivan et al. 2025), have been often analyzed, impulsivity remains relatively unexplored. Impulsivity refers to a behavioral disposition to act rapidly with limited consideration of potential consequences (Moeller et al. 2001). It includes attentional impulsivity (difficulty maintaining concentration), non‐planning impulsivity (limited consideration of future outcomes), and motor impulsivity (acting without thinking; Patton et al. 1995). From a neurobiological point of view, impulsivity has been associated with alterations in the fronto‐striatal and fronto‐insular circuits, which are crucial for inhibitory control and self‐regulation (Dalley et al. 2011; Fineberg et al. 2014). Indeed, in neurodegenerative diseases, such as Parkinson's disease (PD) or frontotemporal dementia, high impulsivity levels have been associated with specific executive dysfunctions (i.e., impaired inhibitory control, reduced cognitive flexibility, and difficulties in monitoring behavior) and represent a behavioral predictor of cognitive decline over time (Mariano et al. 2020; Santangelo, Raimo, and Spitaleri 2017). In MS, emerging evidence has similarly highlighted the clinical relevance of impulsivity. For instance, Toro et al. (2018) reported higher levels of cognitive and motor impulsivity in PwMS compared with healthy controls, while Joly et al. (2020) examined impulsivity as a broader construct, identifying key associations with information‐processing speed, fatigue, and depressive symptoms. However, these pioneering studies did not integrate a detailed, multidimensional characterization of trait impulsivity with a comprehensive assessment of its concurrent cognitive and behavioral correlates and overlooked the potential confounding or explanatory role of dynamic performance fluctuations in PwMS.

Indeed, individual differences in impulsivity in MS could be related to intra‐individual cognitive variability (IICV; Merritt et al. 2019), which quantifies fluctuations in cognitive performance across tasks or within testing sessions. IICV is considered a sensitive indicator of neural inefficiency and cognitive instability in ageing and clinical populations, reflecting deficits in sustained attention and executive monitoring (Hultsch et al. 2008; Merritt et al. 2019). High IICV in MS has been associated with increased white‐matter disruption and greater risk of cognitive decline (Riegler et al. 2021; Arnett et al. 2025), suggesting that cognitive performance can be dynamically modulated by the integrity of executive control systems. Importantly, because both impulsivity and IICV rely on overlapping top‐down regulatory mechanisms, fluctuations in cognitive efficiency could influence the extent to which impulsive tendencies translate into cognitive and behavioral dysfunctions (MacDonald et al. 2009).

The aim of the present study is to provide an integrated neuropsychological and behavioral characterization of impulsivity in PwMS, explicitly considering its multidimensional nature and examining the distinct cognitive and behavioral correlates of attentional, motor, and non‐planning impulsivity. Differently from previous investigations, here impulsivity is considered concurrently with IICV and global cognitive status, thereby addressing a significant gap in the literature and providing a more comprehensive view of top‐down dysregulation in MS. Therefore, the present study aimed at determining if impulsivity assessed by the Barratt Impulsiveness Scale version 11 total (BIS‐11; Stanford et al. 2009) and its sub‐scales (i.e., attentional, motor, and non‐planning) scores is associated with cognitive and behavioral profile in MS, and whether these associations persist after accounting for IICV and global cognitive status.

## 2 Materials and Methods

### 2.1 Participants

Fifty‐four PwMS (43 women, 79.6%) meeting the revised McDonald diagnostic criteria (Thompson et al. 2018) were enrolled at the Department of Psychology, University of Campania “Luigi Vanvitelli”, and at the Sant'Anna e San Sebastiano Hospital, Caserta, Italy. Participants were excluded according to the following criteria: (i) diagnosis of major neurocognitive disorder according to the Diagnostic and Statistical Manual of Mental Disorders, Fifth Edition, Text Revision (DSM‐5‐TR, American Psychiatric Association 2022); (ii) global cognitive impairment defined as an age‐ and education‐adjusted Montreal Cognitive Assessment (MoCA; Nasreddine et al. 2005) score <15.5 according to Italian norms (Santangelo et al. 2015); (iii) severe disability as indicated by a score higher than 7 on the Expanded Disability Status Scale (EDSS; Kurtzke 1983); (iv) history of head trauma, other neurologic diseases, or alcohol or drug abuse; and (v) non‐native Italian speakers.

Clinical information was collected for each participant, including age at disease onset, disease duration, level of disability (EDSS), and current pharmacological treatment. Disease‐modifying therapies were as follows: interferon beta‐1a (*n* = 13), glatiramer acetate (*n* = 2), natalizumab (*n* = 2), fingolimod (*n* = 4), dimethyl fumarate (*n* = 13), teriflunomide (*n* = 6), and no current treatment (*n* = 14). The study was conducted in accordance with the principles of the Declaration of Helsinki and was approved by the Ethics Committee of the University of Campania “Luigi Vanvitelli”, Caserta, Italy. Written informed consent was obtained from all participants prior to study participation.

### 2.2 Neuropsychological Assessment

All participants underwent MoCA to assess global cognitive functioning, adjusted according to the Italian normative criteria (Santangelo et al. 2015), and the Italian version of the Rao's Brief Repeatable Battery of Neuropsychological Tests (BRB‐N; Rao et al. 1991; Amato et al. 2006) to assess specific cognitive domains. The BRB‐N included the following tests:

- Selective Remind Test (SRT; Buschke and Fuld 1974) assessed verbal learning by a list of 12 unrelated words. In the first part of the test, all words are read out to the participants, who are required to remember as many as possible of them. In the subsequent five trials, only the words not recalled in the previous trial are presented. A word that is recalled for two consecutive trials is considered to have entered the long‐term memory store (LTS), and then the total number of LTS words recalled for all six trials is considered (SRT‐LTS). Finally, a word recalled in all subsequent trials is classified as Consistent Long‐Term Retrieval (CLTR). After a delay of 20–25 min, the participants are required to recall as many words as possible from the list; the total number of words recalled after the delay period is rated as Delayed Recall (SRT‐D). The Italian version of BRB‐N does not provide normative values for the cued delayed recall subtest.
- *10/36 Spatial Recall Task* (10/36 SPART; Barbizet and Cany 1968) assessed visuospatial learning by a chessboard with 10 pawns in specific positions. In three consecutive trials, participants are provided an empty chessboard and black plastic checkers and are required to replicate the position of 10 checkers shown on a chessboard for 10 s. After a delay of 20–25 min, the participants are asked again to replicate the stimulus on the empty chessboard. The score is the total number of correct answers for the three tests (SPART) and the delayed recall test (SPART‐D).
- Symbol Digit Modalities Test (SDMT; Smith 1991) assessed visual‐spatial processing speed and working memory. In this task, an 8.5 × 11‐inch sheet of paper with nine symbols, each paired with a number at the top of the page, is provided to the participants; in the rest of the page, a pseudo‐random sequence of these symbols is printed. The participants are required to tell the number corresponding to each symbol in the shortest possible time. The total score is given by the total number of correct answers in 90 s.
- Paced Auditory Serial Addition Task (PASAT; Gronwall 1977) assessed auditory processing speed and working memory. In this task, the participants are auditorily presented with a sequence of 60 digits (digitally recorded) and are asked to add up consecutively each digit to the immediately preceding one. The digits are initially presented at a rate of one every 3 s (PASAT 3), while in the second trial, a 2‐second interval is used (PASAT 2). The total score is given by the total number of correct answers in each trial.
- Word List Generation (WLG; Rao 1990) assessed semantic verbal fluency, by requiring the participants to produce as many words as possible belonging to a semantic category (vegetable and fruit) within 90 s. The total score is given by the number of correct words.
- Stroop word‐color task (ST; Barbarotto et al. 1998) assessed sustained attention and inhibitory control. In the first trial of the task, the participants are required to read a list of words indicating colors printed in black ink as quickly as possible; in the second trial, participants are presented with differently colored dots and have to name the color of the single dots as quickly as possible; in the third trial (interference condition), participants are presented with a list of words indicating colors, each printed in a different color; the participants have to name the ink color of words without reading the words as quickly as possible. The total stimuli were 100, and the total score is the time required to name 50 items in the third trial (higher scores indicate worse performance).

According to Hancock et al. (2024), functionally intact PwMS showing impairment of greater than −1.5 SD below the normative mean (see Amato et al. 2006) on at least two objective neuropsychological measures were classified as having mild cognitive impairment (i.e., MS‐MCI).

The IICV was computed according to previous studies (Rabinowitz and Arnett 2013; Arnett et al. 2025). Raw test scores were standardized as *z*‐scores, and then two variability indices were calculated: the maximum discrepancy score (MDS), the difference between the highest and lowest score *z*‐scores (Rabinowitz and Arnett 2013), and the intraindividual standard deviation (ISD), the standard deviation of z‐scores across tests battery within a specific cognitive domain (Merritt et al. 2019). Consistent with prior literature (Riegler et al. 2021), MDS and ISD were averaged to obtain three domain‐specific IICV indices (see Arnett et al. 2025): memory IICV (SRT and SPART scores), executive IICV (WLG and ST scores), and attention/processing speed IICV (PASAT and SDMT scores).

### 2.3 Behavioral Assessment

The Interoceptive Sensibility was assessed by the Self‐Awareness Questionnaire (SAQ; Longarzo et al. 2015), a self‐report questionnaire composed of 35 items to be rated on a five‐point Likert scale (0 = never; 1 = sometimes; 2 = often; 3 = very often; 4 = always), specifically developed to assess the frequency of common bodily feelings, and how the body signals are felt by respondents. The total score ranges from 0 to 140, with higher scores indicating higher levels of interoceptive sensibility and a focus on one's bodily sensations. Items were grouped into two domains: visceral domain that includes sensations related to autonomic, visceral, and internal bodily states (e.g., “I feel that I can't get enough air into my lungs”; including item 1, 3, 4, 5, 6, 7, 10, 12, 13, 15, 17, 20, 22, 25, 26, 27, 29, 30, 31, 33, 35) and somatosensory domain that reflects bodily sensations generated by musculoskeletal and superficial systems (e.g., “I feel very hot in comparison to others”; including item 2, 8, 9, 11, 14, 16, 18, 19, 21, 23, 24, 28, 32, 34).

The severity of depression symptoms was assessed by the Beck Depression Inventory‐II (BDI‐II; Beck et al. 1996; validated in MS, Sacco et al. 2016), a self‐report questionnaire which consists of 21 items scored on a four‐point Likert scale from a minimum of 0 points to a maximum of 3, with higher scores reflecting higher levels of depression.

The severity of apathy was assessed by the Dimensional Apathy Scale (DAS; Santangelo, Raimo, Trojano, et al. 2017), a self‐report questionnaire validated in MS (Raimo et al. 2020), which evaluated apathy in neurological diseases. The DAS consisted of 24 items scored on a four‐point Likert scale (0 = almost always; 1 = often; 2 = occasionally; 3 = hardly ever). The 24 items were divided into three apathetic subscales: (1) executive subscale (items 1, 6, 10, 11, 17, 19, 21, 23) that evaluated apathy associated with impaired planning, attention, or organization; (2) emotional subscale (items 3, 5, 7, 9, 12, 15, 20, 24) that evaluated emotional integration; and (3) behavioral cognitive initiation subscale (items 2, 4, 8, 13, 14, 16, 18, 22) that evaluated self‐generation of behavior or cognition. The total score ranges from 0 to 72, with higher scores indicating more severe apathy.

The severity of impulsivity was assessed by the BIS‐11 (Stanford et al. 2009), a self‐report 30‐item questionnaire scored on a 4‐point Likert scale (1 = rarely/never, 2 = occasionally, 3 = often, 4 = almost/always). Items *1, 7, 8, 9, 10, 12, 13, 15, 20, 29, 30* were reverse‐scored (1 = almost always/always; 2 = often; 3 = occasionally; 4 = rarely/never) before calculating the subscale and total scores. The questionnaire was divided into three subscales: attentional, motor, and non‐planning impulsivity. Attentional impulsivity (items 5, 6, 9, 11, 20, 24, 26, 28) refers to difficulties in sustaining attention and maintaining focus, often resulting in distractibility or frequent shifting of attention between tasks. Motor impulsivity (items 2, 3, 4, 16, 17, 19, 21, 22, 23, 25, 30) refers to the propensity to act without thinking, often with impulsive, risk‐taking behavior or action taken in great haste. Non‐planning impulsivity (items 1, 7, 8, 10, 12, 13, 14, 15, 18, 27, 29) measures the difficulty in organizing and structuring future‐oriented goals, potentially leading to hasty decisions and poor impulse control in long‐term planning. The total score ranges from 30 to 120, with higher scores indicating higher levels of impulsivity.

### 2.4 Statistical Analysis

Normality of each measure was assessed using the Kolmogorov–Smirnov test; as several variables resulted to be not normally distributed (e.g., SRT‐LTS, SRT‐D, SPART‐D, PASAT‐3, and ST; all *p* < 0.01), non‐parametric analyses were performed. To examine the relationship between impulsivity (i.e., total and subscales) and cognitive variables (i.e., SRT‐LTS, SRT‐CLTR, SRT‐D, SPART, SPART‐D, SDMT, PASAT 2, PASAT 3, WLG, ST, BDI‐II, DAS, and SAQ), Spearman correlation analyses were conducted. To control for multiple comparisons, a Bonferroni correction was applied, with the statistical significance level defined as *p* ≤ 0.002.

Subsequently, on the significant correlations after Bonferroni correction, hierarchical multiple regression analyses were conducted to investigate the relationship between impulsivity, including the total impulsivity score and subscale scores (independent variables, step 1), and the total score of each cognitive or behavioral variables (dependent variables), adjusting for IICV indices (i.e., memory, executive, and attention, Step 2) and global cognitive status (corrected MoCA rank score, Step 3). Given the non‐normal distribution of several variables, a rank transformation was applied before regression modeling. This transformation involved ordering raw scores from lowest to highest and assigning ranks accordingly, thereby reducing the influence of non‐normality and of outliers while preserving the ordinal structure of the data (Iman and Conover 1979).

The strength of associations was assessed using beta coefficients and *p*‐values, while *R* ^2^ values indicated the proportion of variance explained by the predictors.

All analyses were performed using SPSS software (version 21; SPSS Inc., Chicago, IL, USA).

## 3 Results

The demographic and clinical characteristics of the sample are reported in Table 1, whereas the cognitive and behavioral variables are provided in Table 2. Based on disease‐specific cut‐off values previously validated in PwMS (Sacco et al. 2016; Raimo et al. 2020), 18.5% of the participants showed clinically significant depressive symptoms and 22.2% clinically significant apathy. Regarding neurological disability, 70.4% of the participants presented mild disability (EDSS 0–2.5), 16.7% moderate disability (EDSS 3.0–4.5), and 13.0% high disability (EDSS 5.0–7.0). According to the Italian normative values for the BRB‐N and ST (Amato et al. 2006), the proportion of participants scoring below the expected normative range for each neuropsychological measure is reported as follows: SRT‐LTS, 48.1%; SRT‐CLTR, 44.4%; SPART, 16.7%; SDMT, 37.0%; PASAT‐3, 27.8%; SPART‐D, 25.9%; WLG, 42.6%; and ST, 27.8%. Overall, 64.8% of the PwMS met the operational criteria for MS‐MCI, consistent with the prevalence reported in the MS literature (Hancock et al. 2023, 2024).

**TABLE 1:** Mean and standard deviation of demographic, and clinical variables in people with multiple sclerosis (*n* = 54).

Demographic variables	Mean ± SD	Min‐Max
Age (years)	46.80 ± 12.53	21‐75
Education (years)	13.31 ± 3.81	3‐22
Sex (female/male)	43/11	—
Clinical variables		
EDSS	2.69 ± 2.12	0–6.5
Duration of disease (months)	170.20 ± 130.74	1–456
Age at onset of disease (years)	14.65 ± 11.43	1–47
Cognitive variables		
MoCA adjusted score	22.68 ± 2.58	16.52–26.85
Pharmacological treatment	%	—
Dimethyl fumarate	24.1%	—
Fingolimod	7.4%	—
Glatiramer acetate	3.7%	—
Interferon beta‐1a	24.1%	—
Natalizumab	3.7%	—
Teriflunomide	11.1%	—
No treatment	25.9%	—

Abbreviations: EDSS: Expanded Disability Status Scale; MoCA: Montreal Cognitive Assessment; MS: multiple sclerosis.

**TABLE 2:** Neuropsychological assessment in people with multiple sclerosis (*n* = 54).

Cognitive variables	Mean ± SD
Memory	
SRT‐LTS	26.39 ± 14.39
SRT‐CLTR	19.09 ± 13.79
SRT‐D	5.48 ± 3.06
SPART	17.48 ± 5.06
SPART‐D	5.76 ± 2.42
Attention and information processing speed	
SDMT	41.09 ± 13.17
PASAT 3	39.43 ± 15.62
PASAT 2	34.43 ± 12.67
Executive functioning	
WLG	18.48 ± 5.68
ST *(sec)*	100.50 ± 69.92
Behavioral variables	
BIS‐11 Total	58.50 ± 8.89
BIS‐11 Attentional	15.43 ± 3.90
BIS‐11 Motor	18.56 ± 3.65
BIS‐11 Non‐Planning	24.52 ± 4.84
DAS	23.65 ± 8.30
DAS‐executive	8.33 ± 5.12
DAS‐emotional	6.96 ± 3.89
DAS‐cognitive	8.59 ± 4.18
BDI‐II	13.67 ± 8.18
SAQ	35.15 ± 17.34
SAQ‐Visceral	12.67 ± 8.58
SAQ‐Somatosensorial	17.89 ± 7.78

Abbreviations: BDI‐II: Beck Depression Inventory‐II; BIS‐11: Barratt Impulsiveness Scale; DAS: Dimensional Apathy Scale; PASAT‐2: Paced Auditory Serial Addition Test‐2 s; PASAT‐3: Paced Auditory Serial Addition Test‐3 s; SAQ: Self Awareness Questionnaire; SDMT: Symbol Digit Modalities Test; SPART: Spatial Recall Test; SPART‐D: Spatial Recall Test‐Delayed; SRT‐CLTR: Selective Reminding Test‐Consistent Long Term Retrieval; SRT‐D: Selective Reminding Test‐Delayed; SRT‐LTS: Selective Reminding Test‐Long Term Storage; ST: Stroop Test; WLG: Word List Generation.

The results of correlation analyses showed that, after Bonferroni correction (*p* ≤ 0.002), the BIS‐11 total score was not significantly associated with cognitive variables (rho ≤ 0.360; *p* ≥ 0.016, see Table 3). Regarding behavioral variables, the BIS‐11 total score showed moderate and significant correlations with BDI‐II (rho = 0.454; *p* = 0.001), DAS total (rho = 0.595; *p* < 0.001), and DAS executive (rho = 0.446; *p* = 0.001). No other behavioral correlations were significant (rho ≤ 0.393; *p* ≥ 0.003; see Table 3). The BIS‐11 attentional subscale was not significantly correlated with cognitive variables (rho ≤ 0.212; *p* ≥ 0.166; see Table 3), whereas moderate and significant associations were found with SAQ total (rho = 0.405; *p* = 0.002), DAS total (rho = 0.442; *p* = 0.001), DAS executive (rho = 0.467; *p* < 0.001), and BDI‐II (rho = 0.570; *p* < 0.001). No other correlations were found to be statistically significant (rho ≤ 0.370; *p* ≥ 0.006; see Table 3).

**TABLE 3:** Correlations between BIS‐11, cognitive functions, and behavioral domains in people with multiple sclerosis.

Variables		BIS‐11 Total	BIS‐11 Attentional	BIS‐11 Motor	BIS‐11 Non‐Planning
Cognitive variables					
SRT‐LTS	rho	−0.181	0.059	−0.059	−0.320*
	*p*	0.189	0.672	0.673	0.018
SRT‐CLTR	rho	−0.008	0.185	0.036	−0.188
	*p*	0.954	0.181	0.795	0.174
SRT‐D	rho	−0.134	0.049	0.012	−0.323*
	*p*	0.334	0.724	0.934	0.017
SPART	rho	−0.301*	−0.093	−0.233	−0.343*
	*p*	0.027	0.503	0.089	0.011
SPART‐D	rho	−0.183	−0.079	−0.202	−0.168
	*p*	0.184	0.570	0.144	0.224
SDMT	rho	−327*	−0.170	−0.052	−0.430**
	*p*	0.016	0.220	0.709	**0.001**
PASAT 3	rho	−0.347*	−0.036	−0.208	−0.371*
	*p*	0.011	0.801	0.134	0.006
PASAT 2	rho	−0.327*	−0.071	−0.276	−0.329*
	*p*	0.019	0.621	0.050	0.018
WLG	rho	−0.226	−0.023	−0.136	−0.258
	*p*	0.100	0.867	0.327	0.059
ST (Sec)	rho	0.360*	0.212	0.279	0.354*
	*p*	0.016	0.166	0.067	0.018
Behavioral variables					
DAS	rho	0.595**	0.442**	0.155	0.546**
	*p*	**<0.001**	**0.001**	0.223	**<0.001**
DAS‐executive	rho	0.446**	0.467**	0.114	0.329*
	*p*	**0.001**	**<0.001**	0.274	0.015
DAS‐emotional	rho	0.238	0.096	0.105	0.274*
	*p*	0.084	0.489	0.448	0.045
DAS‐cognitive	rho	0.393*	0.229	0.025	0.405**
	*p*	0.003	0.095	0.857	**0.002**
BDI‐II	rho	0.454**	0.570**	0.122	0.269
	*p*	**0.001**	**<0.001**	0.380	0.050
SAQ	rho	0.325*	0.405**	0.193	0.125
	*p*	0.017	**0.002**	0.163	0.369
SAQ‐Visceral	rho	0.274*	0.370*	0.153	0.092
	*p*	0.045	0.006	0.270	0.508
SAQ‐Somatosensorial	rho	0.283*	0.327*	0.216	0.107
	*p*	0.038	0.016	0.117	0.441

Abbreviations: BDI‐II: Beck Depression Inventory‐II; BIS‐11: Barratt Impulsiveness Scale; DAS: Dimensional Apathy Scale; PASAT‐2: Paced Auditory Serial Addition Test‐2 s; PASAT‐3: Paced Auditory Serial Addition Test‐3 s; SAQ: Self Awareness Questionnaire; SDMT: Symbol Digit Modalities Test; SPART: Spatial Recall Test; SPART‐D: Spatial Recall Test‐Delayed; SRT‐CLTR: Selective Reminding Test‐Consistent Long Term Retrieval; SRT‐D: Selective Reminding Test‐Delayed; SRT‐LTS: Selective Reminding Test‐Long Term Storage; ST: Stroop Test; WLG: World List Generation.

*p ≤ 0.05; **p ≤ 0.002 after Bonferroni correction are reported in bold.

The BIS‐11 motor subscale was not significantly associated with either cognitive variables (rho ≤ 0.279; *p* ≥ 0.067) or behavioral variables (rho ≤ 0.216; *p* ≥ 0.117; see Table 3).

The BIS‐11 non‐planning subscale was not significantly correlated with most cognitive variables after Bonferroni correction (rho ≤ 0.371; *p* ≥ 0.006; see Table 3), except for a significant association with SDMT (rho = −0.430; *p* = 0.001). Regarding behavioral variables, the BIS‐11 non‐planning subscale was significantly associated with DAS total (rho = 0.546; *p* < 0.001) and DAS cognitive (rho = 0.405; *p* = 0.002). No other correlations survived Bonferroni correction (rho ≤ 0.329; *p* ≥ 0.015; see Table 3).

A three‐step hierarchical multiple regression analysis was conducted on the Bonferroni‐corrected significant association (*p* ≤ 0.002) between BIS‐11 non‐planning impulsivity and SDMT performance. In Step 1, the BIS‐11 non‐planning rank score significantly predicted SDMT rank score (*F* = 11.784, *p* = 0.001), explaining 18.5% of the variance (*R* ^2^ = 0.185). Higher non‐planning impulsivity was associated with lower SDMT rank score (*β* = −0.430, *t* = −3.433, *p* = 0.001; see Table 4). In Step 2, after adding the IICV‐Attention rank score, the explained variance increased to 30.7% (*F* = 11.306, *p* < 0.001; *R* ^2^ = 0.307). Both BIS‐11 non‐planning impulsivity (*β* = −0.363, *p* = 0.004) and IICV‐Attention (*β* = −0.356, *p* = 0.004) remained significant predictors of SDMT rank score (see Table 4). In Step 3, after adding global cognitive status, indexed by the corrected MoCA rank score, the explained variance increased to 34.7% (*F* = 8.872, *p* < 0.001; *R* ^2^ = 0.347); however, the additional contribution of global cognitive status was not statistically significant (Δ*R* ^2^ = 0.040, *p* = 0.085). In the final model, BIS‐11 non‐planning impulsivity remained significantly associated with lower SDMT rank score (*β* = −0.322, *p* = 0.009), after accounting for IICV‐Attention and global cognitive status (see Table 4). IICV‐Attention also remained a significant predictor (*β* = −0.327, *p* = 0.008), whereas the corrected MoCA rank score did not reach statistical significance (*β* = 0.208, *p* = 0.085; see Table 4).

**TABLE 4:** Hierarchical regression analyses examining the contribution of impulsivity, IICV domains, and global cognitive status to SDMT performance.

Model	Step	Predictor	*β*	*p*	*R* ^2^	Δ*R* ^2^	*p* (Δ*R* ^2^)
Attention	1	BIS‐11 Non‐planning	−0.430	0.001	0.185	—	—
	2	BIS‐11 Non‐planning	−0.363	0.004	0.307	0.122	0.004
		IICV‐Attention	−0.356	0.004	—	—	—
	3	BIS‐11 Non‐planning	−0.322	0.009	0.347	0.040	0.085
		IICV‐Attention	−0.327	0.008	—	—	—
		MoCA score	0.208	0.085	—	—	—
Memory	1	BIS‐11 Non‐planning	−0.430	0.001	0.185	—	—
	2	BIS‐11 Non‐planning	−0.430	0.001	0.185	0.000	0.917
		IICV‐Memory	−0.013	0.917	—	—	—
	3	BIS‐11 Non‐planning	−0.373	0.005	0.246	0.061	0.050
		IICV‐Memory	0.001	0.993	—	—	—
		MoCA score	0.254	0.050	—	—	—
Executive	1	BIS‐11 Non‐planning	−0.430	0.001	0.185	—	—
	2	BIS‐11 Non‐planning	−0.393	0.003	0.226	0.041	0.105
		IICV‐Executive	−0.206	0.105	—	—	—
	3	BIS‐11 Non‐planning	−0.321	0.012	0.304	0.078	0.022
		IICV‐Executive	−0.246	0.048	—	—	—
		MoCA score	0.289	0.022	—	—	—

Abbreviations: β: standardized regression coefficient; Δ*R* ^2^: change in explained variance between Step 1 and Step 2; BIS‐11: Barratt Impulsiveness Scale‐11; IICV: intra‐individual cognitive variability; MoCA: corrected Montreal Cognitive Assessment score; *R* ^2^: proportion of explained variance; SDMT: Symbol Digit Modalities Test.

In the models including IICV‐Memory and IICV‐Executive rank scores, the addition of the respective IICV measure at Step 2 did not significantly increase the explained variance (IICV‐Memory: Δ*R* ^2^ = 0.000, *p* = 0.917; IICV‐Executive: Δ*R* ^2^ = 0.041, *p* = 0.105; see Table 4). At Step 3, the addition of global cognitive status did not significantly increase the explained variance in the IICV‐Memory model (Δ*R* ^2^ = 0.061, *p* = 0.050), whereas it significantly increased the explained variance in the IICV‐Executive model (Δ*R* ^2^ = 0.078, *p* = 0.022). In both final models, BIS‐11 non‐planning impulsivity remained significantly associated with lower SDMT rank score after accounting for the respective IICV measure and global cognitive status (IICV‐Memory: *β* = −0.373, *p* = 0.005; IICV‐Executive: *β* = −0.321, *p* = 0.012; see Table 4). In the IICV‐Executive model, IICV‐Executive also remained a significant predictor of SDMT rank score (*β* = −0.246, *p* = 0.048), whereas the corrected MoCA rank score was significantly associated with SDMT rank score (*β* = 0.289, *p* = 0.022; see Table 4).

Linear regression analyses showed that BIS‐11 total rank score significantly predicted DAS total (*β* = 0.595, *t* = 5.332, *p* < 0.001), DAS executive (*β* = 0.446, *t*  = 3.598, *p* = 0.001), and BDI‐II (*β* = 0.454, *t*  = 3.670, *p* = 0.001). The attentional subscale significantly predicted DAS total (*β* = 0.442, *t* = 3.550, *p* = 0.001), DAS executive (*β* = 0.467, *t*  = 3.812, *p* < 0.001), BDI‐II (*β* = 0.570, *t*  = 5.003, *p* < 0.001), and SAQ total (*β* = 0.405, *t* = 3.190, *p* = 0.002). Finally, the BIS‐11 non‐planning subscale significantly predicted DAS total (*β* = 0.546, *t* = 4.699, *p* < 0.001), and DAS cognitive (*β* = 0.405, *t*  = 3.192, *p* = 0.002).

## 4 Discussion

The present study investigated the relationship between impulsivity and its subdomains with cognitive and behavioral alterations in PwMS, with particular attention to the contribution of IICV and global cognitive status.

Overall, the findings indicate that impulsivity represents a clinically relevant dimension in MS, characterized by specific associations with cognitive efficiency, motivational‐affective disorders, and self‐regulation processes. Importantly, the novelty of the present investigation lies in showing that these associations were not fully explained by IICV or global cognitive status, suggesting that impulsivity captures a complementary and independent dimension of top‐down control dysfunction in MS independently from transient performance fluctuations (Toro et al. 2018). These data help clarify the added value of assessing trait impulsivity alongside traditional objective neuropsychological metrics. Specifically, non‐planning impulsivity was significantly associated with reduced performance on the SDMT, a usually recognized measure of processing speed, sustained attention, working memory, and the efficiency of maintaining task goals over time in MS (Benedict et al. 2012; Kalb et al. 2018). Therefore, higher non‐planning impulsivity may reflect reduced capacity for future‐oriented organization and goal management, which negatively affects performance on tasks requiring rapid integration and continuous rule application (DeLuca et al. 2020).

This pattern is consistent with evidence from other neurodegenerative conditions, such as PD and Alzheimer's disease, in which planning‐related impulsivity has been associated with reduced executive efficiency after accounting for global cognitive status (Dirnberger and Jahanshahi 2013; Aarsland et al. 2017; Rochat et al. 2010). Non‐planning impulsivity was also associated with apathy, particularly its cognitive dimension. This supports neurocognitive models suggesting that both impulsivity and apathy reflect dysfunctions in goal formulation, initiation, and maintenance, mediated by fronto‐striatal circuits (Levy and Dubois 2006; Raimo et al. 2020). Moreover, attentional impulsivity was strongly associated with depressive symptoms and executive apathy. This dimension reflects difficulty maintaining focus and resisting distraction, which may exacerbate mood dysregulation and motivational impairment (Robbins et al. 2012). Regression analyses confirmed that attentional impulsivity independently predicted apathy and depression, underscoring its clinical relevance. The association with interoceptive sensitivity suggests that heightened monitoring of internal bodily states may compete with external task demands, further contributing to attentional instability and affective vulnerability (Craig 2009; Critchley and Harrison 2013; Raimo et al. 2024).

In contrast, motor impulsivity showed no significant associations with cognitive or behavioral variables and could be due to MS mainly affecting white matter integrity and distributed fronto‐parietal networks rather than primary dopaminergic systems primarily linked to motor impulsivity (Rocca et al. 2015).

Some limitations of the present study should be acknowledged. Although the sex distribution of our relatively small sample reflects the epidemiology of MS, the marked female predominance (43 women and 11 men) may limit the generalizability of the findings, particularly to male PwMS. Future studies including more balanced samples are warranted to further investigate potential sex‐related differences. Furthermore, future longitudinal studies are needed to clarify causal relationships between impulsivity, IICV, and cognitive decline. Multimodal neuroimaging would be needed to elucidate the neural correlates of impulsive behavior in MS that were not explored here. Moreover, extending the investigation to progressive phenotypes may reveal stage‐specific patterns.

In conclusion, this study demonstrates that impulsivity, particularly its non‐planning and attentional components, is associated with cognitive inefficiency and motivational‐affective alterations in PwMS independently from IICV and global cognitive status. Impulsivity may therefore represent a clinically meaningful marker of self‐regulatory dysfunction and a potential target for cognitive‐behavioral interventions aimed at improving executive control, emotional regulation, and daily functioning.

## Author Contributions

**Mariachiara Gaita**: conceptualization, methodology, investigation, formal analysis, writing – original draft. **Antonio Costanzo**: conceptualization, data curation, investigation. **Valentina Torchia**: conceptualization, data curation, investigation. **Ylenia Crocetto**: conceptualization, data curation, investigation. **Luigi Trojano**: writing – review and editing. **Gabriella Santangelo**: Writing – review and editing. **Stefania Miniello**: Writing – review and editing. **Simona Raimo**: Conceptualization, methodology, investigation, writing – original draft, writing – review and editing.

## Funding

The authors have nothing to report.

## Ethics Statement

All study procedures were conducted in accordance with the principles of the Declaration of Helsinki and were approved by the Ethics Committee of the University of Campania “Luigi Vanvitelli,” Caserta, Italy.

## Consent

Written informed consent was obtained from all participants prior to study inclusion.

## Conflicts of Interest

The authors declare no conflicts of interest.

## Data Availability

Data are available on request from the authors.
